# Gamma‐synuclein is a novel prognostic marker that promotes tumor cell migration in biliary tract carcinoma

**DOI:** 10.1002/cam4.4121

**Published:** 2021-07-09

**Authors:** Yusuke Takemura, Hidenori Ojima, Go Oshima, Masahiro Shinoda, Yasushi Hasegawa, Minoru Kitago, Hiroshi Yagi, Yuta Abe, Shutaro Hori, Yoko Fujii‐Nishimura, Naoto Kubota, Yuki Masuda, Taizo Hibi, Michiie Sakamoto, Yuko Kitagawa

**Affiliations:** ^1^ Department of Surgery Keio University School of Medicine Tokyo Japan; ^2^ Department of Pathology Keio University School of Medicine Tokyo Japan; ^3^ Department of Pathology International University of Health and Welfare School of Medicine Chiba Japan; ^4^ Department of Pediatric Surgery and Transplantation Kumamoto University Graduate School of Medical Sciences Kumamoto Japan

**Keywords:** biliary tract carcinoma, gamma‐synuclein, immunohistochemistry

## Abstract

Gamma‐synuclein (SNCG) promotes invasive behavior and is reportedly a prognostic factor in a range of cancers. However, its role in biliary tract carcinoma (BTC) remains unknown. Consequently, we investigated the clinicopathological significance and function of SNCG in BTC. Using resected BTC specimens from 147 patients with adenocarcinoma (extrahepatic cholangiocarcinoma [ECC, *n* = 96]; intrahepatic cholangiocarcinoma [ICC, *n* = 51]), we immunohistochemically evaluated SNCG expression and investigated its correlation with clinicopathological factors and outcomes. Furthermore, cell lines with high SNCG expression were selected from 16 BTC cell lines and these underwent cell proliferation and migration assays by siRNAs. In the results, SNCG expression was present in 22 of 96 (22.9%) ECC patients and in 10 of 51 (19.6%) ICC patients. SNCG expression was significantly correlated with poorly differentiated tumor in both ECC and ICC (*p *= 0.01 and 0.03, respectively) and with perineural invasion and lymph node metastases in ECC (*p* = 0.04 and 0.003, respectively). Multivariate analyses revealed that SNCG expression was an independent poor prognostic factor in both OS and RFS in both ECC and ICC. In vitro analyses showed high SNCG expression in three BTC cell lines (NCC‐BD1, NCC‐BD3, and NCC‐CC6‐1). Functional analysis revealed that SNCG silencing could suppress cell migration in NCC‐BD1 and NCC‐CC6‐1 and downregulate cell proliferation in NCC‐CC6‐1 significantly. In conclusion, SNCG may promote tumor cell activity and is potentially a novel prognostic marker in BTC.

## Graphical abstract

This study revealed for the first time the clinicopathological roles of gamma‐synuclein (SNCG) in biliary tract carcinoma (BTC). SNCG was identified as a strong independent poor prognostic factor of overall and recurrence‐free survival and was also significantly correlated with poor differentiation and perineural invasion in BTC.

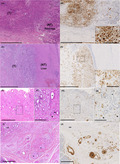

## 1 INTRODUCTION

Biliary tract carcinoma (BTC) comprises malignant epithelial tumors with features of cholangiocyte differentiation arising from liver, large bile ducts, or gallbladder. BTC is known as having one of the poorest prognoses, and the disease incidence has been increasing.^1^, ^2^, ^3^ Surgical resection is the only curative treatment for BTC, but many cases are unresectable at the time of diagnosis; moreover, most patients suffer the recurrence of BTC after curative surgery. Extensive local invasion and a high frequency of lymph node metastases at diagnosis also contribute to poor outcomes.^4^, ^5^, ^6^ Consequently, identifying new molecules associated with invasiveness or metastasis could improve the prognosis as an early diagnostic marker or constitute a new therapeutic target. However, the detailed pathological mechanism of the development and progression of BTC remains ill‐defined.

Gamma‐synuclein (SNCG), a member of the synuclein family, is a small, soluble protein that is highly expressed in neuronal tissues.^7^ Moreover, we previously found that SNCG is strongly expressed in pancreatic cancer cells.^8^ Further clinicopathological study revealed that SNCG expression in pancreatic cancer tissues was significantly correlated with invasive features (such as perineural invasion, lymphovascular invasion, or lymph node metastases) and was an independent predictor of poor prognosis in pancreatic cancer.^9^ BTC has many common features with pancreatic cancer, for example, tissue type, primary site, high invasiveness, and dismal prognosis.^10^, ^11^ Against this background, SNCG is suggested to be critically involved in the invasiveness and prognosis of BTC. However, in BTC, there is no evidence indicating the clinicopathological role of SNCG. Consequently, in the present study, we investigated the clinicopathological significance and function of SNCG in BTC using immunohistochemical and in vitro approaches.

## 2 MATERIALS AND METHODS

### 2.1 Patients

Among the 153 patients who underwent curative surgical resection for extrahepatic cholangiocarcinoma (ECC) or intrahepatic cholangiocarcinoma (ICC) at Keio University hospital between January 2000 and December 2016, 147 (96 ECC cases and 51 ICC cases) were included in the current study. Patients with other synchronous malignant diseases (*n* = 2), patients whose tissue samples were unavailable (*n* = 2), and patients who did not give written informed consent (*n* = 2) were excluded. We diagnosed ECC (including perihilar and distal cholangiocarcinoma) or ICC according to the World Health Organization (WHO) classification 2019.^12^ The curability was evaluated by the eighth edition of the TNM classification of malignant tumors published by the Union for International Cancer Control (UICC).^13^ Histologic diagnoses of vascular and lymphatic invasion were made according to the sixth edition of the Japanese general rules for clinical and pathological studies on cancer of the biliary tract.^14^ The definition and degree of perineural invasion were determined as described previously.^8^ Clinical and pathological data were obtained from patients’ medical records. We performed radical surgery to achieve R0 resection with regional lymph node dissection. Patients with mass‐forming ICC underwent hepatic resection without lymph node dissection if clinically there was no lymph node metastasis. As a result, pancreatoduodenectomy (PD), extrahepatic bile duct resection (EHBD), hepatectomy with extrahepatic bile duct resection (Hx+EHBD), hepatectomy (Hx), and combined hepatectomy and pancreatoduodenectomy (HPD) were performed in 46, 12, 59, 26, and 4 patients, respectively. After surgical resection, follow‐up examinations were performed using computed tomography and measurement of the serum carcinoembryonic antigen (CEA) and carbohydrate antigen 19‐9 (CA19‐9) levels every 3 or 6 months. Tumor recurrence was diagnosed as tumor growth at any site of the body after surgery using clinical and radiological investigations. The median follow‐up for censored cases was 38.9 months (range, 4.0–151.4 months).

### 2.2 Cell lines

Experiments were performed using 16 BTC cell lines. Thirteen cell lines were previously established from Japanese patients with BTC^15^, ^16^, ^17^: NCC‐BD1, NCC‐BD2, NCC‐BD3, NCC‐BD4‐1, and NCC‐BD4‐2 were derived from ECC, and NCC‐CC1, NCC‐CC3‐1, NCC‐CC3‐2, NCC‐CC4‐1, NCC‐CC4‐2, NCC‐CC5, NCC‐CC6‐1, and NCC‐CC6‐2 were derived from ICC. Moreover, we used three BTC cell lines (TKKK, OZ, and HuCCT1) purchased from Riken BioResource Center or from the Japanese Collection of Research Bioresources. OZ and HuCCT1 were derived from ECC, whereas TKKK was derived from ICC. TKKK and OZ cells were cultivated in DMEM plus 10% FBS and antibiotics, whereas the remaining 14 cell lines were cultivated in RPMI‐1640 medium plus 10% FBS and antibiotics. NCC‐BD2, NCC‐BD3, NCC‐BD4‐1, NCC‐CC1, NCC‐CC3‐1, NCC‐CC6‐1, and TKKK cells were cultured on collagen I‐coated dishes. The remaining nine cell lines were cultivated on normal culture dishes. All cells were kept at 37℃ in an atmosphere of 5% CO_2_.

### 2.3 Immunohistochemical staining and evaluation

Immunohistochemical staining was performed on both the cell lines and the resected tumors. Resected specimens were fixed in 10% formalin and embedded in paraffin. Cell blocks of the cultivated cell lines were also fixed in 10% formalin and embedded in HoldGel110 (ASIAKIZAI Co., Ltd.). Both were then cut into 4‐µm thick slices for staining. Immunohistochemical staining using mouse anti‐human SNCG monoclonal antibody (1:150 dilution; 1H10D2; Santa Cruz Biotechnology) was performed using a Leica Bond‐Max automated immunostainer (Leica Microsystems) according to the manufacturer’s instructions. Peripheral nerves and vascular endothelial cells consistently showed intense SNCG staining and therefore served as internal positive controls. We defined a case as SNCG positive if more than 10% of tumor cells had cytoplasmic staining, as described in our previous report.^9^ The selection of representative sections, histological diagnosis, and the evaluation of immunohistochemical staining were independently conducted by two researchers (TY and HO) without reference to the clinicopathological data. If the initial evaluations yielded different results, a consensus interpretation was reached after re‐examination.

### 2.4 Quantitative real‐time PCR

RNA isolation was performed according to the manufacturers’ instructions using an RNeasy Mini Kit (Qiagen) to extract RNA from six‐well plates, and a SuperPrep II Cell Lysis & RT kit for qPCR (TOYOBO) to extract RNA from 96‐well plates. For reverse transcription of RNA to DNA, a ReverTra Ace qPCR RT Kit (TOYOBO) was used according to the manufacturer’s instructions. Subsequently, qPCR was performed on a Step One Plus Real‐Time PCR System (Applied Biosystems). A PrimeTime qPCR Probe (Integrated DNA Technologies) for the *SNCG* (Hs.PT.58.20259255) gene was utilized for the quantitative analysis of mRNA transcript levels. The *GAPDH* gene was used as an internal control (Applied Biosystems, NM_002046.3). The specific gene expression levels were calculated using the ΔΔCt method. Assays were performed in triplicate.

### 2.5 SNCG knockdown for cell lines

A total of 5 × 10^5^ or 2 × 10^4^ cells, respectively, were seeded on 6‐well or 96‐well plates and transfected with either 7.5 or 0.3 pg of non‐target small interfering RNA (Silencer Select Negative Control No. 1 siRNA Cat#4390843, Thermo Fisher Scientific) or with 7.5 or 0.3 pg of siRNA against human SNCG (siSNCG‐1: s194807 and siSNCG‐2: s194805, Thermo Fisher Scientific). Lipofectamine RNAiMAX Transfection Reagent (Thermo Fisher Scientific) was used for transfection, according to the manufacturer’s instructions. The cells were processed for further analysis after 48 h of siRNA transfection.

### 2.6 Immunoblotting analysis

For protein extraction, cultured cells were homogenized with 200 µl of RIPA lysis buffer (Thermo Fisher Scientific), 2 µl of proteinase inhibitor (Nacalai Tesque), and 2 µl of phosphatase inhibitor cocktail (Nacalai Tesque). After centrifuging (15,000 rpm, 10 min), supernatants were collected. Total protein was quantified using Protein Assay CCB Solution (Nacalai Tesque). Twenty micrograms of protein was fractionated with sample buffer solution with reducing reagent for SDS‐PAGE (Nacalai Tesque) on acrylamide gel (Mini‐Protean TGX Precast Gel, Bio‐Rad) and electro‐transferred to nitrocellulose membranes. After blocking with 5% skim milk for 1 h, the membranes were probed with antibodies against SNCG (1:200 dilution; 1H10D2, Santa Cruz Biotechnology) or β‐actin (1:500 dilution; C4, Santa Cruz Biotechnology) overnight in a cold room. Peroxidase‐conjugated anti‐mouse antibody (1:5000 dilution; NA931‐1ML, GE Healthcare Life Sciences) was used as a secondary antibody and signals were enhanced by highly sensitive detection substrate (Chemi‐Lumi One Super, Nacalai Tesque).

### 2.7 Cell proliferation assay

In total, 5 × 10^3^ cells were seeded onto 96‐well plates. After 24 h, siRNA was added and after a further 72 h, cell viability was measured using CellTiter‐Glo reagent (Promega) according to the manufacturer’s instructions. Assays were performed in triplicate and three independent assays were performed.

### 2.8 Wound healing assay

Cell migration was assessed by wound healing assay using an IncuCyte Zoom Kinetic Live Cell Imaging System (Essen BioScience). Ninety‐six‐well ImageLock Plates (Essen BioScience) were coated overnight with 300 μg/ml of 3‐D Culture Matrix Collagen I (Cat# 3447‐020‐01, R&D systems). The following day, collagen I dilution was aspirated, and cells were plated in six replicates to form fully confluent monolayers. Four hours after seeding, scratches were made with a 96‐pin WoundMaker (Essen Bioscience). Cells were covered in 100 μl of media. Migration was quantified using the Relative Wound Density metric calculated using IncuCyte software. Three separate assays were conducted.

### 2.9 Statistical analysis

Data are given as the mean ± standard deviation (SD) or the number of patients (%). Intergroup comparisons were performed using Student’s *t‐*test or the *χ* ^2^ test for continuous and categorical variables, respectively. Macroscopic mass‐forming ICC is generally recognized as small duct‐type cholangiocarcinoma.^18^ Consequently, macroscopic mass‐forming ICC rarely invades nerve tissues because of the anatomical distances involved. Therefore, we divided ICC cases into mass‐forming and non‐mass‐forming types to analyze the correlation between SNCG and perineural invasion more rigorously. Overall survival (OS) and recurrence‐free survival (RFS) were calculated using the Kaplan–Meier method and the log‐rank test was applied to compare survival curves. To identify risk factors for survival, we performed Cox hazard proportional univariate and multivariate analyses. The variables included were sex, age, tumor marker, diabetes mellitus, liver cirrhosis, hepatic viral status, surgical procedure, tumor location, differentiation, invasion to other organs, invasion to major vessels (including portal vein, hepatic artery, and inferior vena cava), perineural invasion, lymphatic invasion, vascular invasion, lymph node metastases, curability, and SNCG expression. For ECC cases, the depth of invasion was included and for ICC cases, the macroscopic type and tumor size were included. Based on a previous report, we included carcinoma in situ on the edge of the bile duct in the curative resection group.^19^ Variables with *p *< 0.05 in the univariate analyses, variables which were associated clinicopathologically with SNCG expression, and clinically important variables identified in previous reports (ECC: lymph node metastasis, invasion to other organs, depth of invasion, and curability; ICC: lymph node metastasis and curability) were included in the multivariate analyses to control for confounding effects.^20^, ^21^, ^22^, ^23^ Missing values were not imputed. *p *< 0.05 was considered statistically significant. SPSS 25.0 statistical software (IBM Corp.) was used to perform all the statistical calculations.

## 3 RESULTS

### 3.1 Correlation between SNCG overexpression and clinicopathological parameters

**FIGURE 1:**
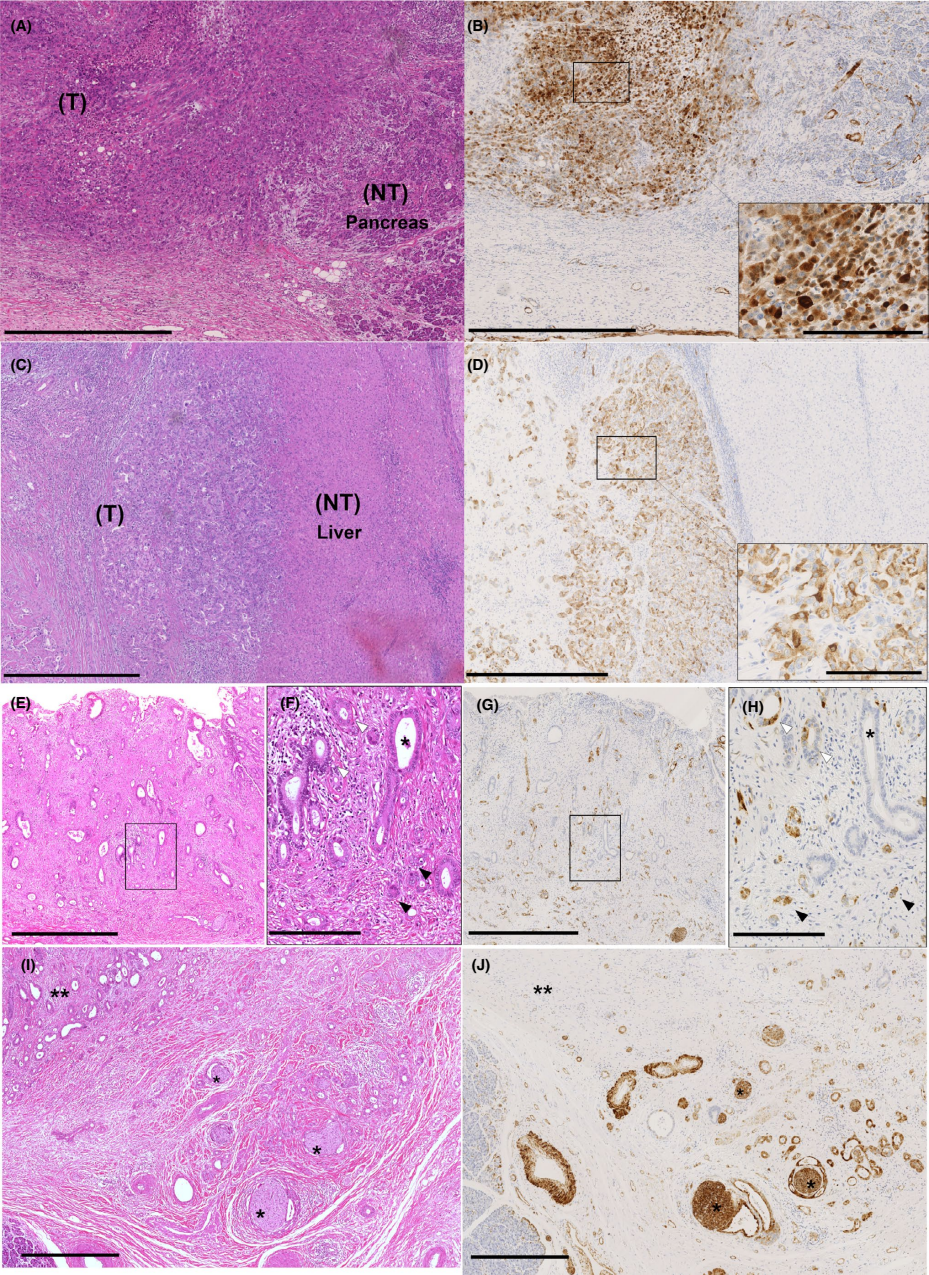
Immunohistochemical expression of gamma‐synuclein (SNCG) in surgically resected specimens. (A–D) Representative SNCG‐positive cases of extrahepatic cholangiocarcinoma (ECC) (A, B) and ICC (C, D) are shown (H&E staining [A, C] and immunohistochemical staining of SNCG [B, D]). The immunohistochemical expression of SNCG was observed in most of the tumor cells (T: tumor). Moreover, peripheral nerves and vascular endothelial cells consistently showed intense SNCG staining and therefore served as internal positive controls. However, there was no positive staining in other non‐tumor areas including stromal cells and surrounding organs (NT: non‐tumor background of pancreas [A, B] and liver [C, D]). The cytoplasmic expression of SNCG was observed in tumor cells (B, D inset). Scale bars represent 1.0 mm in lower magnification views and 200 µm in higher magnification views. (E–H) Representative invasive ECC cases are shown. There was a tendency for tumor cells at the invasive front to more strongly express SNCG than those at the center or superficial regions. The patient was diagnosed with moderately differentiated adenocarcinoma, but adenocarcinoma of various degrees of differentiation was observed (*: well differentiated, white arrowheads: moderately differentiated, black arrowheads: poorly differentiated, E, F: H&E staining, G, H: SNCG staining; F and H correspond to the boxed areas in E and G, respectively). The degree of differentiation decreased as the tumor invaded and SNCG expression was observed in moderately to poorly differentiated adenocarcinomas, but almost no SNCG expression was observed in well‐differentiated adenocarcinomas. Scale bars indicate 1.0 mm in lower magnification views (E, G) and 200 µm in higher magnification views (F, H). (I, J) SNCG expression in one of the patients with neural invasion (I: H&E staining, J: SNCG staining). In this patient, tumor in areas with neural (*) invasion showed stronger SNCG expression than tumor in areas away from neural invasion (**). Scale bars indicate 500 µm

Figure 1 shows representative SNCG staining images of the resected specimens. The immunohistochemical expression of SNCG was observed in the cytoplasm of the tumor cells (T: tumor). There was no positive staining in other non‐tumor areas including fibrous stroma and surrounding organs without peripheral nerves and vascular endothelial cells. Positive immunohistochemical expression of SNCG (Figure 1A–D) was found in 22 of 96 (22.9%) ECC patients and in 10 of 51 (19.6%) ICC patients. The clinicopathological differences between the SNCG‐positive groups and SNCG‐negative groups are summarized in Table 1. SNCG positivity was correlated with poorly differentiated tumor (Figure 1E–H) in both ECC and ICC (*p* = 0.01 and 0.03, respectively). For ECC, perineural invasion (grade 2 and 3) (Figure 1I,J) and lymph node metastasis were associated with SNCG expression (77.3% vs. 52.7%, *p* = 0.04 and 63.6% vs. 28.4%, *p *= 0.003, respectively); however, there was no such association for ICC.

**TABLE 1:** Clinicopathological correlations associated with gamma‐synuclein (SNCG) expression

Variables	ECC (*n *= 96)			ICC (*n* = 51)		
	SNCG (+) (*n* = 22)	SNCG (−) (*n* = 74)	*p*	SNCG (+) (*n* = 10)	SNCG (−) (*n* = 41)	*p*
Male	18 (81.8)	55 (74.3)	0.47	6 (60.0)	29 (70.7)	0.51
Age (years)	68.3 ± 9.1	67.7 ± 9.6	0.78	69.8 ± 11.3	65.0 ± 10.2	0.20
CEA (ng/ml)	3.8 ± 4.2	3.0 ± 1.6	0.39	13.2 ± 28.5	7.5 ± 14.4	0.37
CA19‐9 (ng/ml)	1127.5 ± 4.774.8	124.4 ± 230.9	0.34	1840.1 ± 5456.0	777.5 ± 3167.0	0.42
Diabetes mellitus	4 (18.2)	20 (28.2)	0.35	2 (20.0)	7 (17.5)	0.85
Liver cirrhosis	—	—		1 (10.0)	4 (9.8)	0.98
Hepatic viral status	—	—		2 (20.0)	11 (27.5)	0.63
Primary lesion						
Distal	15 (68.2)	46 (62.2)	0.61	—	—	
Perihilar	7 (31.8)	28 (37.8)		—	—	
Surgical resection			0.51			0.95
PD	13 (59.1)	33 (44.6)		—	—	
EHBD	2 (9.1)	10 (13.5)		—	—	
Hx+EHBD	7 (31.8)	27 (36.5)		5 (50.0)	20 (48.8)	
Hx	—	—		5 (50.0)	21 (51.2)	
HPD	0 (0.0)	4 (5.4)		—	—	
Depth of invasion					
Carcinoma in situ or invasion to fibromuscular layer	0 (0.0)	6 (8.1)	0.25	—	—	
Invasion into subserosa	15 (68.2)	53 (71.6)		—	—	
Beyond serosal invasion	7 (31.8)	15 (20.3)		—	—	
Mass forming + periductal infiltrating type	—	—		1 (10.0)	8 (19.5)	0.48
Tumor size (cm)	—	—		6.2 ± 4.2	5.0 ± 2.9	0.28
**Differentiation**						
**Well**	**1 (4.5)**	**27 (36.5)**	**0.01**	**0 (0)**	**11 (26.8)**	**0.03**
**Moderate**	**13 (59.1)**	**36 (48.6)**		**8 (80.0)**	**28 (68.3)**	
**Poor**	**8 (36.4)**	**11 (14.9)**		**2 (20.0)**	**1 (2.4)**	
**Unclassified**	**0 (0)**	**0 (0)**		**0 (0.0)**	**1 (2.4)**	
Invasion to other organs	9 (40.9)	17 (23.0)	0.10	0 (0.0)	3 (7.3)	0.38
**Perineural invasion**					
**0–1**	**5 (22.7)**	**35 (47.3)**	**0.04**	8 (80.0)	28 (68.3)	0.47
**2–3**	**17 (77.3)**	**39 (52.7)**		2 (20.0)	13 (31.7)	
Lymphatic invasion						
0–1	10 (45.5)	43 (58.1)	0.30	8 (80.0)	33 (80.5)	0.97
2–3	12 (54.5)	31 (41.9)		2 (20.0)	8 (19.5)	
Vascular invasion					
0–1	8 (36.4)	42 (56.8)	0.09	8 (80.0)	35 (85.4)	0.68
2–3	14 (63.6)	32 (43.2)		2 (20.0)	6 (14.6)	
Invasion to major vessels	1 (6.3)	13 (24.5)	0.11	4 (40.0)	14 (34.1)	0.73
**Lymph node metastasis**	**14 (63.6)**	**21 (28.4)**	**0.003**	5 (50.0)	13 (31.7)	0.28
R1 resection	3 (13.6)	17 (23.0)	0.34	1 (10.0)	5 (12.2)	0.85
Adjuvant therapy	12 (54.5)	26 (35.1)	0.11	5 (50.0)	16 (39.0)	0.61

Note

Data are presented as mean values ± SDs for continuous variables and numbers (%) for categorical variables. Bold emphasis indicates statistical significance (*p* < 0.05).

Abbreviations: CA19‐9, carbohydrate antigen 19‐9; CEA, carcinoembryonic antigen; EHBD, extrahepatic bile duct resection; HPD, combined hepatectomy and pancreatoduodenectomy; Hx, hepatectomy; Hx+EHBD, hepatectomy with extrahepatic bile duct resection; NCG, gamma‐synuclein; PD, pancreatoduodenectomy.

Furthermore, to evaluate the association between SNCG expression and perineural invasion more accurately, we divided ICC cases into mass‐forming and non‐mass‐forming groups (Table S1). Analysis based on this division showed that there was no correlation with SNCG and perineural invasion in the non‐mass‐forming ICC group (50.0% vs. 35.3%, *p *= 0.68).

### 3.2 Prognostic impact of SNCG overexpression on BTC

**FIGURE 2:**
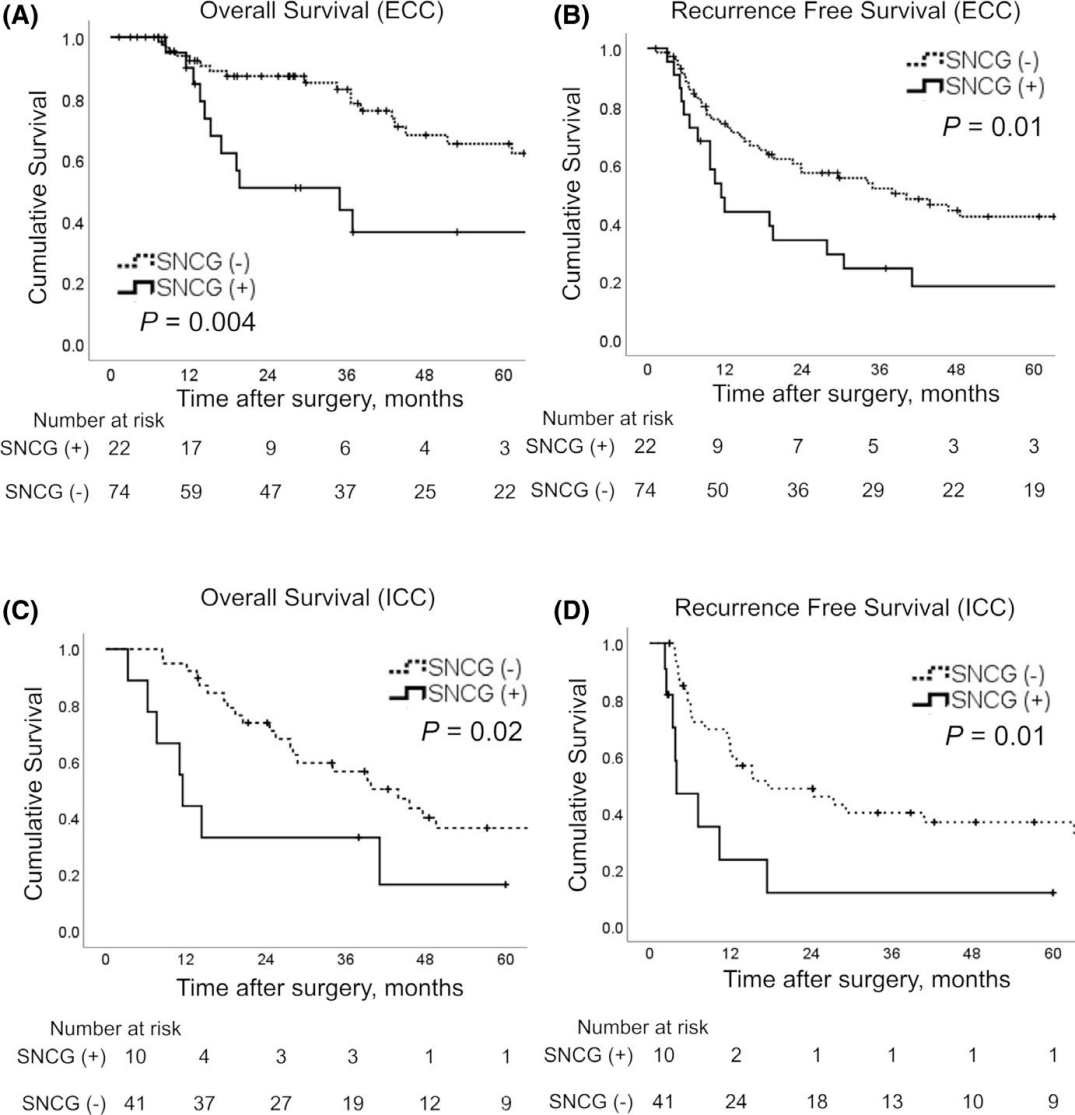
Survival curves according to gamma‐synuclein (SNCG) expression. Patients with SNCG overexpression (solid lines) had a significantly poorer prognosis than patients with negative SNCG expression (dashed lines) in terms of overall and recurrence‐free survival in extrahepatic cholangiocarcinoma (ECC) (A, B) and intrahepatic cholangiocarcinoma (ICC) (C, D)

The 3‐year OS rate was 73.8% for ECC and 52.5% for ICC. The SNCG‐positive group demonstrated significantly reduced OS and RFS compared to the SNCG‐negative group for both ECC (median OS, 35.0 months vs. not reached, respectively, *p* = 0.01 and median RFS, 11.5 vs. 40.2 months, respectively, *p *= 0.01) and for ICC (median OS, 11.5 vs. 43.9 months, respectively, *p* = 0.02, and median RFS, 4.0 vs. 17.6 months, respectively, *p *= 0.01) (Figure 2).

Cox hazard proportional univariate and multivariate analyses were performed to investigate whether SNCG expression affected prognosis independently. The results of univariate analyses are summarized in Tables 2 and 3. Univariate analyses revealed that patients with SNCG‐positive tumors had poorer prognoses than patients with SNCG‐negative tumors for both ECC and ICC. Multivariate analysis of ECC identified SNCG expression (HR, 2.6; 95% CI, 1.1–6.2; *p *= 0.03), HPD (HR, 7.6; 95% CI, 1.2–46.5; *p *= 0.03, PD as reference), and CA19‐9 (HR, 2.9; 95% CI, 1.1–7.7; *p *= 0.03) as independent prognostic factors for OS and also identified SNCG expression (HR, 1.9; 95% CI, 1.0–3.7; *p *= 0.04), EHBD (HR, 3.0; 95% CI 1.1–8.0; *p *= 0.03, PD as reference), and lymphatic invasion (HR, 2.3; 95% CI, 1.1–4.7; *p *= 0.02) as independent prognostic factors for RFS. Multivariate analysis of ICC identified SNCG expression (HR, 3.6; 95% CI, 1.2–11.2; *p *= 0.03), age ≥70 years (HR, 2.7; 95% CI, 1.1–7.0; *p* = 0.04), and lymphatic invasion (HR, 9.2; 95% CI, 1.7–51.1; *p *= 0.01) as independent prognostic factors for OS and also identified SNCG expression (HR, 3.6; 95% CI, 1.2–10.5; *p* = 0.02) and lymphatic invasion (HR, 5.2; 95% CI, 1.1–25.0; *p *= 0.04) as independent prognostic factors for RFS (Table 4).

**TABLE 2:** Univariate analysis of the prognostic factors for extrahepatic cholangiocarcinoma

			Overall survival			Recurrence‐free survival	
		HR	95% CI	*p*	HR	95% CI	*p*
Sex	Male	1.6	0.8–3.4	0.19	1.1	0.6–2.0	0.81
	Female	1.0			1.0		
Age (years)	**≥**70	1.9	0.9–4.1	0.12	1.6	0.8–3.0	0.15
	<70	1.0			1.0		
CEA (ng/ml)	**≥**5	0.8	0.2–3.6	0.82	1.3	0.6–3.1	0.50
	<5	1.0			1.0		
**CA19‐9 (ng/ml)**	**≥37**	**3.7**	**1.7–8.1**	**0.001**	**1.9**	**1.1–3.3**	**0.02**
	**<37**	**1.0**			**1.0**		
Diabetes mellitus	Present	0.8	0.3–1.9	0.57	1.0	0.5–1.8	0.92
	Absent	1.0			1.0		
Location	Perihilar	1.6	0.8–3.3	0.20	1.4	0.8–2.5	0.19
	Distal	1.0			1.0		
**Surgical procedure**	**PD**	**1.0**			**1.0**		
	**EHBD**	**1.7**	**0.6–4.7**	**0.34**	**1.7**	**0.8–3.6**	**0.18**
	**Hx+EHBD**	**1.8**	**0.8–3.9**	**0.16**	**1.5**	**0.8–2.7**	**0.20**
	**HPD**	**8.2**	**1.7–39.8**	**0.01**	**4.6**	**1.3–16.1**	**0.02**
Differentiation	Poorly differentiated	1.5	0.7–3.5	0.33	1.8	0.9–3.3	0.07
	Others	1.0			1.0		
**Depth of invasion**	**Invasion into subserosa or beyond bile duct wall**	4.0	0.9–17.6	0.06	**3.3**	**1.2–9.2**	**0.02**
	**Carcinoma in situ or invasion to fibromuscular layer**	1.0			**1.0**		
Invasion to other organs	Positive	1.9	0.9–4.0	0.09	1.7	0.9–2.9	0.09
	Negative	1.0			1.0		
Invasion to major vessels	Positive	1.3	0.5–3.3	0.53	1.1	0.5–2.4	0.81
	Negative	1.0			1.0		
**Perineural invasion**	**2–3**	**3.9**	**1.7–8.8**	**0.001**	**2.4**	**1.4–4.4**	**0.002**
	**0–1**	**1.0**					
**Lymphatic invasion**	**2–3**	**3.3**	**1.6–7.1**	**0.002**	**3.3**	**1.9–5.8**	**<0.001**
	**0–1**	**1.0**					
**Vascular invasion**	**2–3**	**3.6**	**1.7–7.5**	**0.001**	**2.5**	**1.4–4.3**	**0.001**
	**0–1**	**1.0**					
**Lymph node metastasis**	**Positive**	**2.3**	**1.1–4.6**	**0.02**	**2.0**	**1.2–3.4**	**0.01**
	**Negative**	**1.0**			**1.0**		
Curability	R1 resection	**2.2**	**1.03–4.7**	**0.04**	1.3	0.7–2.4	0.49
	R0 resection	**1.0**			1.0		
Adjuvant therapy	Present	1.0	0.5–2.0	0.95	1.1	0.6–1.9	0.79
	Absent	1.0			1.0		
**SNCG**	**Positive**	**2.7**	**1.3–5.6**	**0.01**	**2.2**	**1.2–3.8**	**0.01**
	**Negative**	**1.0**			**1.0**		

Bold emphasis indicates statistical significance (*p* < 0.05).

Abbreviations: CA19‐9, carbohydrate antigen 19–9; CEA, carcinoembryonic antigen; CI, confidential interval; EHBD, extrahepatic bile duct resection; HPD, combined hepatectomy and pancreatoduodenectomy; HR, hazard ratio; Hx+EHBD, hepatic resection with extrahepatic bile duct resection; PD, pancreatoduodenectomy; SNCG, gamma‐synuclein.

**TABLE 3:** Univariate analysis of the prognostic factors for intrahepatic cholangiocarcinoma

			Overall survival			Recurrence‐free survival	
		HR	95% CI	*p*	HR	95% CI	*p*
Sex	Male	1.1	0.7–1.6	0.68	1.0	0.7–1.4	0.81
	Female	1.0			1.0		
**Age (years)**	**≥70**	**2.3**	**1.01–5.1**	**0.047**	**2.5**	**1.2–5.3**	**0.02**
	**<70**	**1.0**			**1.0**		
CEA (ng/ml)	**≥**5	1.4	0.6–3.2	0.42	1.5	0.7–3.2	0.27
	<5	1.0			1.0		
**CA19–9 (ng/ml)**	**≥37**	**2.3**	**1.1–4.7**	**0.03**	1.9	0.95–3.8	0.07
	**<37**	**1.0**			1.0		
Diabetes mellitus	Present	1.1	0.4–2.9	0.83	1.0	0.4–2.7	0.93
	Absent	1.0			1.0		
Liver cirrhosis	Present	1.5	0.5–4.3	0.45	1.0	0.6–4.6	0.39
	Absent	1.0			1.0		
Hepatic virus	Present	0.4	0.1–1.1	0.07	0.4	0.2–1.1	0.08
	Absent	1.0			1.0		
Surgical procedure	Hx+EHBD	0.7	0.3–1.5	0.35	0.9	0.4–1.7	0.65
	Hx	1.0			1.0		
Macroscopic type	MF + PI	1.4	0.6–3.5	0.45	1.2	0.5–2.9	0.61
	Others	1.0			1.0		
Tumor size (cm)	**≥**5	2.3	0.8–6.7	0.12	1.6	0.8–3.3	0.17
	<5	1.0			1.0		
Differentiation	Poor	2.9	0.4–23.5	0.31	0.9	0.1–6.3	0.87
	Others	1.0			1.0		
Invasion to other organs	Positive	0.6	0.1–4.2	0.57	1.1	0.3–4.8	0.85
	Negative	1.0			1.0		
Macrovascular invasion	Positive	2.1	0.99–4.3	0.052	1.6	0.8–3.2	0.21
	Negative	1.0			1.0		
Perineural invasion	2–3	1.6	0.7–3.4	0.26	1.4	0.6–3.0	0.37
	0–1	1.0			1.0		
**Lymphatic invasion**	**2–3**	**3.8**	**1.6–8.8**	**0.002**	**3.6**	**1.6–8.2**	**0.002**
	**0–1**	**1.0**			**1.0**		
**Vascular invasion**	**2–3**	**2.7**	**1.01–7.2**	**0.047**	1.7	0.6–4.3	0.31
	**0–1**	**1.0**			1.0		
**Lymph node metastasis**	**Positive**	**3.6**	**1.7–7.7**	**0.001**	**3.0**	**1.5–6.1**	**0.002**
	**Negative**	**1.0**			**1.0**		
**Curability**	**R1 resection**	**4.7**	**1.5–14.3**	**0.007**	2.3	0.8–6.8	0.13
	**R0 resection**	**1.0**			1.0		
Adjuvant therapy	Present	1.5	0.7–3.2	0.28	1.8	0.9–3.6	0.11
	Absent	1.0			1.0		
**SNCG**	**Positive**	**2.6**	**1.1–6.2**	**0.03**	**2.9**	**1.3–6.6**	**0.009**
	**Negative**	**1.0**			**1.0**		

Bold emphasis indicates statistical significance (*p* < 0.05).

Abbreviations: CA19‐9, carbohydrate antigen 19‐9; CEA, carcinoembryonic antigen; CI, confidential interval; HR, hazard ratio; Hx, hepatectomy; Hx+EHBD, hepatectomy with extrahepatic bile duct resection; MF + PI, mass forming with periductal infiltrating; SNCG, gamma‐synuclein.

**TABLE 4:** Multivariate analysis of the prognostic factors for extrahepatic cholangiocarcinoma (ECC) and intrahepatic cholangiocarcinoma (ICC)

	Overall survival			Recurrence‐free survival		
	Adjusted HR	95% CI	*p*	Adjusted HR	95% CI	*p*
ECC (*n* = 96) SNCG						
Positive	2.6	1.1–6.2	0.03	1.9	1.0–3.7	0.04
Negative	1.0			1.0		
ICC (*n* = 51) SNCG						
Positive	3.6	1.2–11.2	0.03	3.6	1.2–10.5	0.02
Negative	1.0			1.0		

Note:

For ECC, we adjusted for CA19‐9, surgical procedure, tumor differentiation, depth of invasion, invasion to other organs, perineural invasion, lymphatic invasion, vascular invasion, lymph node metastasis, and curability.

For ICC, we adjusted for age, CA19‐9 (only for overall survival), tumor differentiation, lymphatic invasion, vascular invasion (only for overall survival), lymph node metastasis, and curability.

Abbreviations: CA19‐9, carbohydrate antigen 19‐9; CI, confidential interval; HR, hazard ratio; SNCG, gamma‐synuclein.

### 3.3 Selection of SNCG‐overexpressing cell lines

**FIGURE 3:**
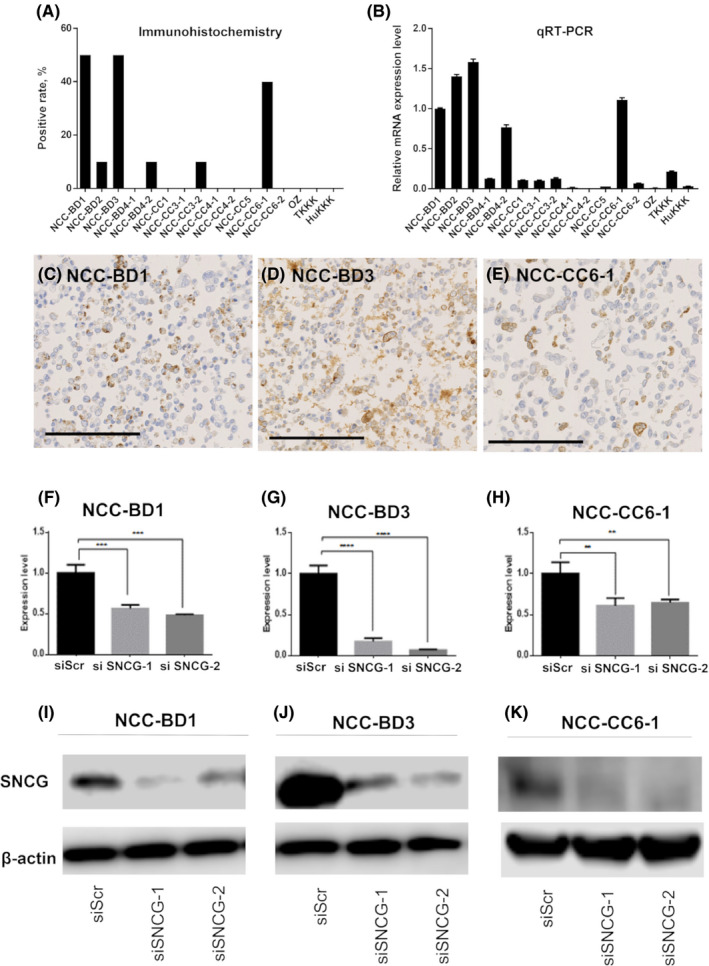
Gamma‐synuclein (SNCG) expression in 16 biliary tract carcinoma (BTC) cell lines and the effects of SNCG knockdown on cell lines that overexpressed SNCG. (A) Immunohistochemical expression rate of SNCG in each BTC cell line. The SNCG positive rates are high in NCC‐BD1, NCC‐BD3, and NCC‐CC6‐1. (B) Real‐time qPCR analysis of SNCG mRNA for each cell line. mRNA expression levels of SNCG were also high in NCC‐BD1, NCC‐BD3, and NCC‐CC6‐1. Levels of SNCG mRNA expression were normalized to those of GAPDH as the internal control. Fold change values with respect to NCC‐BD1 cells shown in the histogram are means ± SDs calculated from three independent experiments. (C) NCC‐BD1, (D) NCC‐BD3, and (E) NCC‐CC6‐1 all highly expressed SNCG. Scale bars indicate 200 µm. (F–H) Inhibition of SNCG expression by siRNA. Cell lines with SNCG overexpression, that is, NCC‐BD1, NCC‐BD3, and NCC‐CC6‐1, were knocked down significantly in terms of SNCG mRNA compared to si‐negative control‐treated cells (siScr). Levels of SNCG mRNA expression were normalized to those of GAPDH as the internal control. Fold change values shown with respect to control cells (siScr) are means ± SDs calculated from three independent experiments (**p < 0.01; ***p < 0.001, ****p < 0.0001). (I–K) Immunoblotting analysis of SNCG in the three cell lines after 48‐h siRNA treatment. β‐actin served as the loading control

To evaluate SNCG expression in each of 16 cell lines, immunohistochemical (Figure 3A) and qPCR analyses (Figure 3B) were performed. Immunohistochemical analyses revealed that only NCC‐BD1, NCC‐BD3, and NCC‐CC6‐1 expressed SNCG in more than 10% of tumor cells (Figure 3C–E). qPCR findings confirmed that these three cell lines strongly expressed SNCG. Therefore, we selected NCC‐BD1, NCC‐BD3, and NCC‐CC6‐1 for further analyses. Next, we knocked down SNCG expression in these three cell lines using siRNA. SNCG expressions were suppressed significantly at both the mRNA and protein levels (Figure 3F–K).

### 3.4 Proliferation and wound healing assays

**FIGURE 4:**
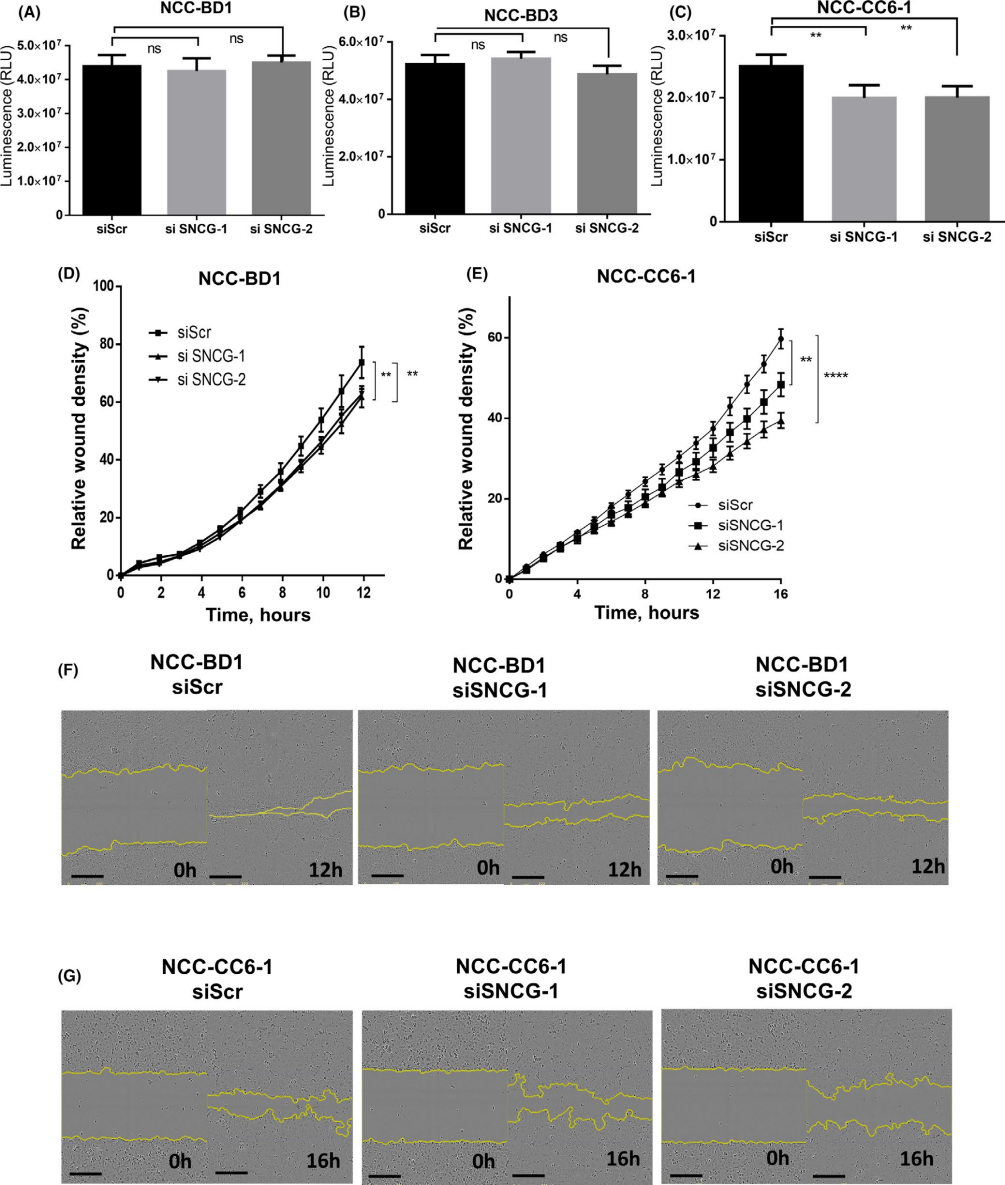
Cell proliferation and wound healing assays. (A–C) Cell proliferation assays were performed with gamma‐synuclein (SNCG)‐overexpressing cell lines NCC‐BD1, NCC‐BD3, and NCC‐CC6‐1. Cells were seeded onto 96‐well plates. After 24 h, siRNA was added, and, after a further 72 h, cell viability was measured. SNCG knockdown by siRNA against human SNCG (siSNCG‐1 and siSNCG‐2) had no effect on the proliferation of NCC‐BD1 or NCC‐BD3 cells compared to si‐negative control‐treated cells (siScr), but SNCG knockdown did suppress the proliferation of NCC‐CC6‐1 cells. Each assay was replicated in three wells. Shown are the means ± SDs of representative data from three independent assays. *p* values were calculated using Student’s *t*‐test. **p < 0.01. (D–G) Cell migration was assessed by wound healing assays using an IncuCyte Zoom Kinetic Live Cell Imaging system. Cells treated with siRNA were plated in six replicates into 96‐well plates coated with collagen I. Four hours after seeding, scratches were made. SNCG knockdown downregulated the migration of NCC‐BD1 (D) and NCC‐CC6‐1 (E) cells significantly compared to si‐negative control‐treated cells. Data plots show the means ± SEs. *p* values were calculated using Student’s *t*‐test at 12 h after making the scratch for NCC‐BD1 cells and at 16 h after making the scratch for NCC‐CC6‐1 cells. **p < 0.01, ****p < 0.0001. Representative figures showing wound healing of NCC‐BD1 (F) and NCC‐CC6‐1 (G) cells. Scale bars indicate 300 µm. The graphs and pictures are representative graphs of three independent experiments

In the proliferation assay, NCC‐BD1 and NCC‐BD3 cells showed no significant difference between control cells and SNCG‐knocked‐down cells. In contrast, NCC‐CC6‐1 cells demonstrated that SNCG knockdown downregulated proliferation significantly (Figure 4A–C).

In wound healing assays, SNCG knocked‐down NCC‐BD1 and NCC‐CC6‐1 cells migrated significantly slower than control cells (Figure 4D–G). NCC‐BD3 cells were excluded from this assay because the attachment between cells was weak and they could not maintain the shape of the scratch wound.

## 4 DISCUSSION

The synuclein family comprises small, soluble proteins that are categorized into alpha, beta, and gamma subgroups. Alpha‐synuclein is expressed in presynaptic neural cells and plays a role in neurotransmission. Moreover, alpha‐synuclein is known to be a key molecule involved in neural degenerative diseases such as familial Parkinson’s disease.^24^ SNCG shares 60% sequence homology with alpha‐synuclein and is also mainly expressed in presynaptic nerve terminals in normal tissues; however, SNCG is also characteristically expressed in malignant tumor cells.^25^ This finding first emerged in studies of advanced breast cancer, but the function and role of SNCG in malignancy are not fully understood. Our previous immunohistochemical study on pancreatic cancer showed a strong correlation between SNCG expression and poor prognosis. However, there are no reports about the clinicopathological correlations between SNCG and BTC, a cancer type that shares several similarities with pancreatic cancer. The present study first clarified detailed clinicopathological correlations between SNCG expression and ECC and ICC, the two major subtypes of BTC. We found that SNCG expression in BTC tumor cells was significantly correlated with poor differentiation and poor prognosis. To support these results, we performed in vitro assays, and these showed that SNCG expression was correlated with tumor cell migration in both ECC and ICC.

Our study revealed strong correlations between SNCG expression and poor differentiation in both ECC and ICC and between SNCG expression and perineural invasion in ECC. We also microscopically examined positive cases in detail. The immunohistochemical expression of SNCG tended to be stronger in poorly differentiated tumor cells than in well‐differentiated tumor cells. SNCG also tended to be more strongly expressed in the invasive area than in the superficial or central portion. And, interestingly, in some cases, immunohistochemical expression of SNCG was more strongly observed in perineural tumor cells than in other areas.

Our previous study on pancreatic cancer also showed that SNCG expression was correlated with perineural invasion.^9^ There are many reported embryological and histological similarities between BTC and pancreatic cancer.^26^, ^27^ They both show frequent perineural invasion and have several molecular markers and somatic mutations in common.^28^, ^29^ Our studies, therefore, suggest that SNCG may have a common function that promotes advanced neural invasion in both ECC and pancreatic cancer. However, in ICC, especially in mass‐forming ICC, perineural invasion is less frequent in general. This likely contributed to the finding that there was no correlation between SNCG expression and perineural invasion in ICC.

Moreover, the current study revealed a strong correlation between SNCG expression and poor differentiation, although several other studies have reported no significant correlation between SNCG expression and differentiation in other cancer types, including pancreatic cancer.^9^, ^30^, ^31^, ^32^, ^33^ Regardless of such clinicopathological diversity, in most cancer types, SNCG expression is correlated with poor prognosis.^9^, ^31^, ^34^, ^35^, ^36^, ^37^ We also identified SNCG expression as an independent poor prognostic factor in both ECC and ICC. Therefore, our data suggest that SNCG may have important roles in promoting the malignant aspects of BTC, as it does in other types of cancers; however, the detailed functions of SNCG likely differ depending on the cancer type and localization.

To evaluate whether SNCG plays a role in the malignancy of BTC, we performed functional assays with BTC cell lines. In migration assays, SNCG silencing significantly decreased cell migration in cell lines from both ECC and ICC. This finding was consistent with previous reports in breast cancer, hepatocellular carcinoma, and gallbladder carcinoma.^38^, ^39^, ^40^ The results of in vitro assays suggested that the role of SNCG may be associated with cell migration, which is generally linked to cell differentiation and metastasis regardless of the origin of the biliary tract tumor. The above‐mentioned findings support the results of our clinicopathological and prognostic analyses. Moreover, our cell proliferation assay data showed that NCC‐CC6‐1, which was derived from ICC, showed significant effects of siRNA interference, whereas the two cell lines from ECC showed no such effects. It has been reported that SNCG increases cell proliferation in gallbladder, colorectal, and prostate cancer.^30^, ^38^, ^41^ However, other studies on breast cancer and pancreatic cancer showed no significant correlation between SNCG expression and cell proliferation.^9^, ^39^ These inconsistencies may result from differences in the functions of SNCG expression in each cancer. In BTC, it is known that gene mutations and expressions differ depending on the location of the tumor, for example, those located in the extrahepatic or intrahepatic bile ducts, and this will likely affect the function of SNCG.^42^ However, it was difficult to compare the functions of SNCG between ICC and ECC in the current study. Although we intended to carry out this analysis using other types of ICC cells, the number of SNCG‐expressing ICC cell lines was limited. This was a limitation of the current study. Further analyses are required.

In recent years, SNCG has been detected in the urine of patients with bladder carcinoma and in the blood serum of patients with gastrointestinal carcinoma.^43^ ^,44^ Some studies also found that anti‐tumor drugs that target SNCG were effective in enhancing the treatment of breast cancer and ectopic endometrium in in vitro and in vivo models.^45,46^ It has also been reported that targeted therapies against the pathways associated with SNCG can control SNCG‐expressing tumors.^39^ ^,47^ In the current study, SNCG expression in resected specimens was identified as a new poor prognostic marker for BTC; moreover, our in vitro model suggested that targeting therapies against SNCG might be effective against SNCG‐expressing BTC. If SNCG can be detected in other sources, such as blood serum or bile acid, it would help inform decisions on the optimal treatment or follow‐up after surgical resection. Consequently, further investigations in in vivo models and preoperative specimens are warranted.

In conclusion, we showed for the first time that the clinicopathological roles of SNCG in BTC include promoting perineural invasion in ECC and promoting poor differentiation in both ECC and ICC. Our functional analysis revealed that SNCG promotes cell migration, a result that was compatible with our clinicopathological findings. We suggest that SNCG has potential as a novel prognostic marker in BTC, but further research is necessary to confirm this.

## CONFLICT OF INTEREST

The authors have no conflict of interest.

## ETHICAL APPROVAL STATEMENT

This study was performed in accordance with the Declaration of Helsinki, subsequent to approval from the institutional review board of Keio University School of Medicine (#20040034) and the National Cancer Center (#2007‐022). Patients gave written informed consent when possible. This study received approval without written informed consent if consent was impossible to obtain.

## Supporting information

Table S1

## Data Availability

The data that support the findings of this study are available from the corresponding author upon reasonable request.
