# Supplementary material for: Gamma‐synuclein is a novel prognostic marker that promotes tumor cell migration in biliary tract carcinoma
Source: Cancer Med. 2021 Jul 9;10(16):5599–613. doi: 10.1002/cam4.4121 (PMC8366101; doi:10.1002/cam4.4121)
Supplement: Supplementary file 1 — Table S1 [file CAM4-10-5599-s001.docx]

**Table S1.** Clinicopathological correlations associated with SNCG expression for mass-forming ICC and non-mass-forming ICC.

|  | Mass-forming ICC (n = 32) | | |  | Non-mass-forming ICC (n = 19) | | |
| --- | --- | --- | --- | --- | --- | --- | --- |
|  | SNCG (+) | SNCG (-) |  |  | SNCG (+) | SNCG (-) |  |
| Variables | (n = 8) | (n = 24) | *P* |  | (n = 2) | (n = 17) | *P* |
| Male | 5 (62.5) | 16 (66.7) | 0.83 |  | 1 (50.0) | 13 (76.5) | 0.42 |
| Age (years) | 68.1± 12.2 | 61.8 ± 9.2 | 0.13 |  | 76.5 ± 0.71 | 70.0 ± 10.0 | 0.38 |
| CEA (ng/mL) | 15,2 ± 31.8 | 4.0 ± 4.5 | 0.09 |  | 5.0 ± 5.5 | 12.7 ± 21.4 | 0.63 |
| CA19-9 (ng/mL) | 125.3± 198.4 | 120.3± 402.7 | 0.97 |  | 8700 ± 12247.8 | 1722.1 ± 4853. | 0.12 |
| Diabetes mellitus | 2 (25.0) | 6 (26.1) | 0.95 |  | 0 (0.0) | 1 (5.9) | 0.73 |
| Liver cirrhosis | 1 (12.5) | 3 (12.5) | 1.00 |  | 0 (0.0) | 1 (5.9) | 0.73 |
| Hepatic viral status | 2 (25.0) | 9 (39.1) | 0.47 |  | 0 (0.0) | 2 (11.8) | 0.61 |
| Macroscopic type |  |  |  |  |  |  |  |
| MF | 8 (100) | 24 (100) | - |  | - | - | - |
| MF+PI | - | - | - |  | 1 (50.0) | 8 (47.1) |  |
| MF+IG | - | - | - |  | 0 (0.0) | 1 (5.9) |  |
| PI | - | - | - |  | 1 (50.0) | 3 (17.6) |  |
| IG | - | - | - |  | 0 (0.0) | 2 (11.7) |  |
| PI+IG | - | - | - |  | 0 (0.0) | 2 (11,7) |  |
| Unclassified |  |  |  |  | 0 (0.0) | 1 (5.9) |  |
| Surgical resection |  |  |  |  |  |  |  |
| Hx+EHBD | 3 (37.5) | 8 (33.3) | 0.83 |  | 2 (100.0) | 12 (70.6) | 0.37 |
| Hx | 5 (62.5) | 16 (66.7) |  |  | 0 (0.0) | 5 (29.4) |  |
| Tumour size **≥** 5 cm | 4 (50.0) | 8 (34.8) | 0.45 |  | 1 (50.0) | 6 (35.2) | 0.73 |
| Differentiation |  |  |  |  |  |  |  |
| Well | 0 (0.0) | 6 (25.0) | 0.09 |  | 0 (0.0) | 5 (29.4) | 0.37 |
| Moderate | 6 (75.0) | 16 (66.7) |  |  | 2 (100.0) | 12 (70.6) |  |
| Poor | 2 (25.0) | 1 (4.2) |  |  | 0 (0.0) | 0 (0.0) |  |
| Unclassified | 0 (0.0) | 1 (4.2) |  |  | 0 (0.0) | 0 (0.0) |  |
| Invasion to other organs | 0 (0.0) | 2 (8.3) | 0.40 |  | 0 (0.0) | 1 (5.9) | 0.73 |
| Perineural invasion |  |  |  |  |  |  |  |
| 0-1 | 7 (87.5) | 22 (91.7) | 0.73 |  | 1 (50.0) | 11 (64.7) | 0.68 |
| 2-3 | 1 (12.5) | 2 (8.3) |  |  | 1 (50.0) | 6 (35.3) |  |
| Lymphatic invasion |  |  | 0.22 |  |  |  | 0.31 |
| 0-1 | 6 (75.0) | 22 (91.7) |  |  | 2 (100.0) | 11 (64.7) |  |
| 2-3 | 2 (25.0) | 2 (8.3) |  |  | 0 (0.0) | 6 (35.3) |  |
| Vascular invasion |  |  | 0.40 |  |  |  | 0.55 |
| 0-1 | 7 (87.5) | 23 (95.8) |  |  | 1 (50.0) | 12 (70.6) |  |
| 2-3 | 1 (12.5) | 1 (4.2) |  |  | 1 (50.0) | 5 (29.4) |  |
|  |  |  |  |  |  |  |  |
| Invasion to major vessels | 3 (37.5) | 8 (33.3) | 0.83 |  | 1 (50.0) | 6 (35.3) | 0.68 |
| Lymph node metastasis | 3 (37.5) | 5 (20.8) | 0.35 |  | 2 (100.0) | 8 (47.1) | 0.16 |
| R1 resection | 0 (0.0) | 2 (8.3) | 0.40 |  | 1 (50.0) | 3 (17.6) | 0.29 |

Data are presented as mean values ± standard deviations for continuous variables and numbers (%) for categorical variables. SNCG gamma-synuclein, CEA carcinoembryonic antigen, CA19-9 carbohydrate antigen 19-9, MF mass forming, PI periductal infiltrating, IG intraductal growth, Hx+EHBD hepatectomy with extrahepatic bile duct resection, Hx hepatectomy.
